# Protective Effect of Koumine, an Alkaloid from Gelsemium Sempervirens, on Injury Induced by H_2_O_2_ in IPEC-J2 Cells

**DOI:** 10.3390/ijms20030754

**Published:** 2019-02-11

**Authors:** Zhihang Yuan, Zengenni Liang, Jine Yi, Xiaojun Chen, Rongfang Li, Yong Wu, Jing Wu, Zhiliang Sun

**Affiliations:** 1 Department of Clinical Veterinary Medicine, College of Veterinary Medicine, Hunan Agricultural University, Changsha 410128, China; zhyuan2016@hunau.edu.cn (Z.Y.); yijine@gmail.com (J.Y.); s51857176@gmail.com (X.C.); wztg_19@163.com (R.L.); ywwz_19@163.com (Y.W.); 2 Hunan Co-Innovation Center for Utilization of Botanical Functional Ingredients, Changsha 410128, China; 3 Department of Hunan Agricultural Product Processing Institute, Changsha 410128, China; enni_007@163.com

**Keywords:** koumine, IPEC-J2 cells, H_2_O_2_, apoptosis

## Abstract

Medicinal herbal plants have been commonly used for intervention in different diseases and improvement of health worldwide. Koumine, an alkaloid monomer found abundantly in *Gelsemium* plants, can be effectively used as an antioxidant. The purpose of this study was to evaluate the potential protective effect of koumine against hydrogen peroxide (H_2_O_2_)-induced oxidative stress and apoptosis in porcine intestinal epithelial cell line (IPEC-J2 cells). MTT assays showed that koumine significantly increased cell viability in H_2_O_2_-mediated IPEC-J2 cells. Preincubation with koumine ameliorated H_2_O_2_-medicated apoptosis by decreasing reactive oxygen species (ROS) production, and efficiently suppressed the lactate dehydrogenase (LDH) release and malondialdehyde (MDA) production. Moreover, a loss of superoxide dismutase (SOD), catalase (CAT) and glutathione (GSH) activities was restored to normal level in H_2_O_2_-induced IPEC-J2 cells upon koumine exposure. Furthermore, pretreatment with koumine suppressed H_2_O_2_-mediated loss of mitochondrial membrane potential, caspase-9 and caspase-3 activation, decrease of Bcl-2 expression and elevation of Bax expressions. Collectively, the results of this study indicated that koumine possesses the cytoprotective effects in IPEC-J2 cells during exposure to H_2_O_2_ by suppressing production of ROS, inhibiting the caspase-3 activity and influencing the expression of Bax and Bcl-2. Koumine could potentially serve as a protective effect against H_2_O_2_-induced apoptosis.

## 1. Introduction

The small intestine is particularly vulnerable to damage induced by endotoxin, and this damage may be involved in plasma and intracellular production of reactive oxygen species (ROS), resulting in cell apoptosis, reducing antioxidative capacity and mitochondrial dysfunction [1,2]. The intestinal epithelium, the border between the body and the environment, is the main place to transport the nutrient. In addition, the enterocyte is the main target of harmful factors and stress, for example, toxin and ROS [3]. Oxidative stress generated by an imbalance between ROS and antioxidants contributes to the pathogenesis of arthritis, cancer, cardiovascular, liver, and respiratory diseases [4]. ROS can cause oxidative damage to macromolecules resulting in lipid peroxidation, oxidation of amino acids, formation of protein–protein cross-links and protein fragmentation, DNA damage, and DNA strand breaks. High concentrations of ROS are cytotoxic leading to necrotic cell death, whereas pretreatment of cells with sublethal ROS levels activates antioxidant defense and/or repair systems, leading to temporary adaptation to oxidative stress [5]. Previous studies indicated that extracellular H_2_O_2_ increased intracellular ROS levels via multiple mechanisms including loss of intracellular ROS antioxidants such as glutathione (GSH) or decreased mitochondrial membrane permeability followed by mitochondrial ROS release [6,7]. Extracellular H_2_O_2_ has been used to induce oxidative injury in intestinal epithelium at different concentration [8,9]. Therefore, H_2_O_2_ was used to induce oxidative injury in IPEC-J2 cells in our study.

To prevent and counteract these effects, the employment of antioxidant molecules is crucial. However, the use of pharmaceutical drugs is sometimes associated with side effects. For this reason, the search for natural alternatives originating has gained increasing attention [5]. *Gelsemium elegans* Benth (*G. elegans*) is a species of the family Longaniaceae widely distributed in southern and over southeastern Asia. Alkaloids, iridoids and several other compounds from a wide spectrum of secondary metabolite classes have been found in *G. elegans.* Koumine is a kind of alkaloid that forms the major active components of *G. elegans*. Studies showed that koumine has a notable potential as an anxiolytic [10] and analgesic [11] agent. In a previous study, we found that koumine inhibits the production of cytokines and the synthesis and secretion of inflammatory mediators by regulating the nuclear factor-κB (NF-κB), p38 and mitogen-activated protein kinase (MAPK)/extracellular signal-regulated kinase (ERK) signaling pathway, thereby exhibiting a potent anti-inflammatory effect [12]. However, the antioxidant effects and mechanisms of koumine are still not clearly understood. Although the studies cited above have shown that *G. elegans* possesses a potent anti-inflammatory effect, whether or not koumine is able to relieve or inhibit the oxidative stress-induced inflammatory response and the specific mechanisms of action of koumine have not been reported. In the present study, IPEC-J2 cells were used to establish a model of H_2_O_2_-induced injury. The protective effects of various concentrations of koumine against H_2_O_2_-induced injury in IPEC-J2 cells were examined at different time points. The present study provides an experimental basis for the clinical application of koumine.

## 2. Results

### 2.1. The Effects of Various Concentrations of H_2_O_2_ on the Viability of IPEC-J2 Cells at Different Time Periods

**Figure 1:**
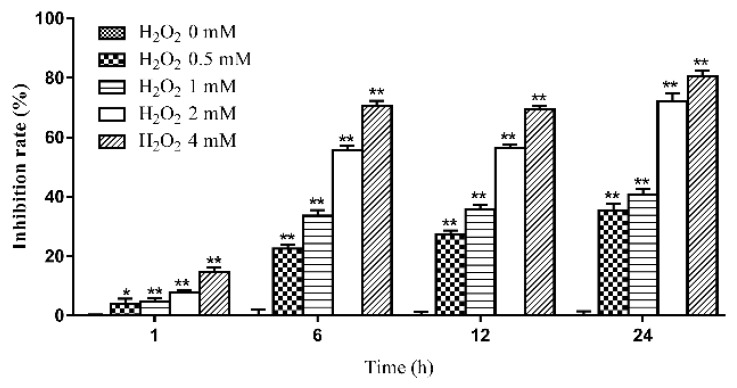
Effect of H_2_O_2_ on the viability of IPEC-J2 cells (mean ± s.d., *n* = 5). Legend: * and ** indicate level of significance at *p* < 0.05 and *p* < 0.01, respectively, compared with the oxidative stress model group.

At high concentrations, H_2_O_2_ induced oxidative stress damage in IPEC-J2 cells and reduced the survival of IPEC-J2 cells. The effect of H_2_O_2_ on IPEC-J2 cells is shown in Figure 1. It was found that the viability of IPEC-J2 cells was reduced after treatment with 0.5 mM H_2_O_2_ for 1, 6, 12 or 24 h (1 h, *p* < 0.05; 6, 12 and 24 h, *p* < 0.01). Based on the above findings, 0.5 mM H_2_O_2_ was used to establish the model of oxidative stress in the present study. The duration of H_2_O_2_ treatment was 1, 6 or 12 h.

### 2.2. The Effects of Various Concentrations of Koumine on the Viability of IPEC-J2 Cells at Different Time Periods

**Figure 2:**
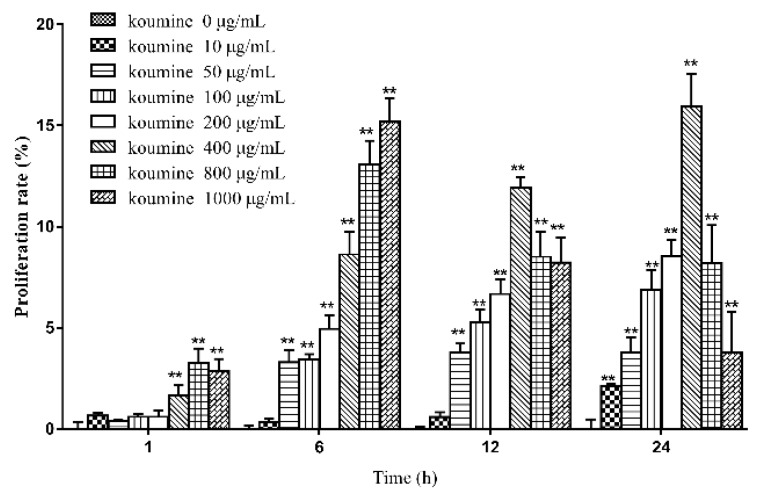
Effect of koumine on the viability of IPEC-J2 cells (mean ± s.d., *n* = 5). Legend: compared with the control group; * and ** indicate level of significance at *p* < 0.05 and *p* < 0.01, respectively, compared with the oxidative stress model group.

Compared with the control group, exposure to 50, 100 or 200 μg/mL koumine increased the viability of IPEC-J cells at various time periods. The increase in cell viability was statistically significant at 6, 12 and 24 h. No significant difference was observed in cell viability when incubated with 10, 50, 100 and 200 μg/mL koumine at 1 h. Cell viability of IPEC-J cells was highest when exposure to 400 μg/mL koumine at 24 h. The results are shown in Figure 2.

### 2.3. Investigation of the Dose-Time-Effect Relationship in Koumine-Mediated Protection against H_2_O_2_-Induced Damage in IPEC-J2 Cells

**Figure 3:**
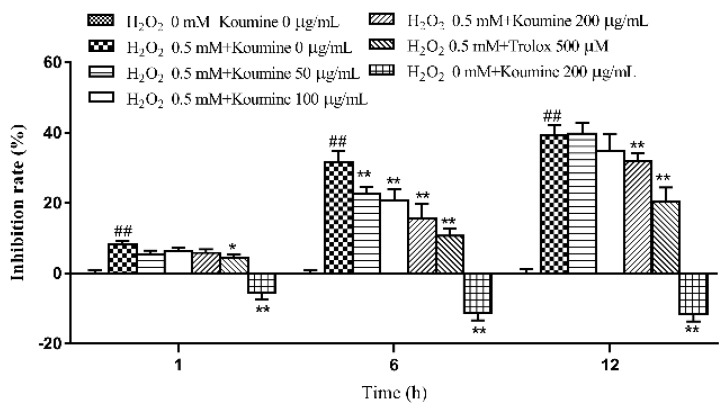
Protective effect of koumine on the viability of IPEC-J2 cells exposed to H_2_O_2_ (mean ± s.d., *n* = 5) Legend: ## indicate level of significance at *p* < 0.01, respectively, compared with the control group; * and ** indicate level of significance at *p* < 0.05 and *p* < 0.01, respectively, compared with the oxidative stress model group.

Compared with the control group, cell viability decreased in the model groups at 1, 6 and 12 h. The decrease in cell viability was statistically significant. Compared with the model group, pretreatment with koumine for 12 h followed by treatment with H_2_O_2_ for 1, 6 or 12 h inhibited the H_2_O_2_-mediated reduction in IPEC-J2 cell viability. Moreover, koumine exerted its inhibitory effect in a time- and dose-dependent manner. The results are shown in Figure 3.

### 2.4. The Effects of Koumine on the LDH Level, Antioxidant Enzyme Activities and MDA Content of H_2_O_2_-Treated IPEC-J2 Cells

**Figure 4:**
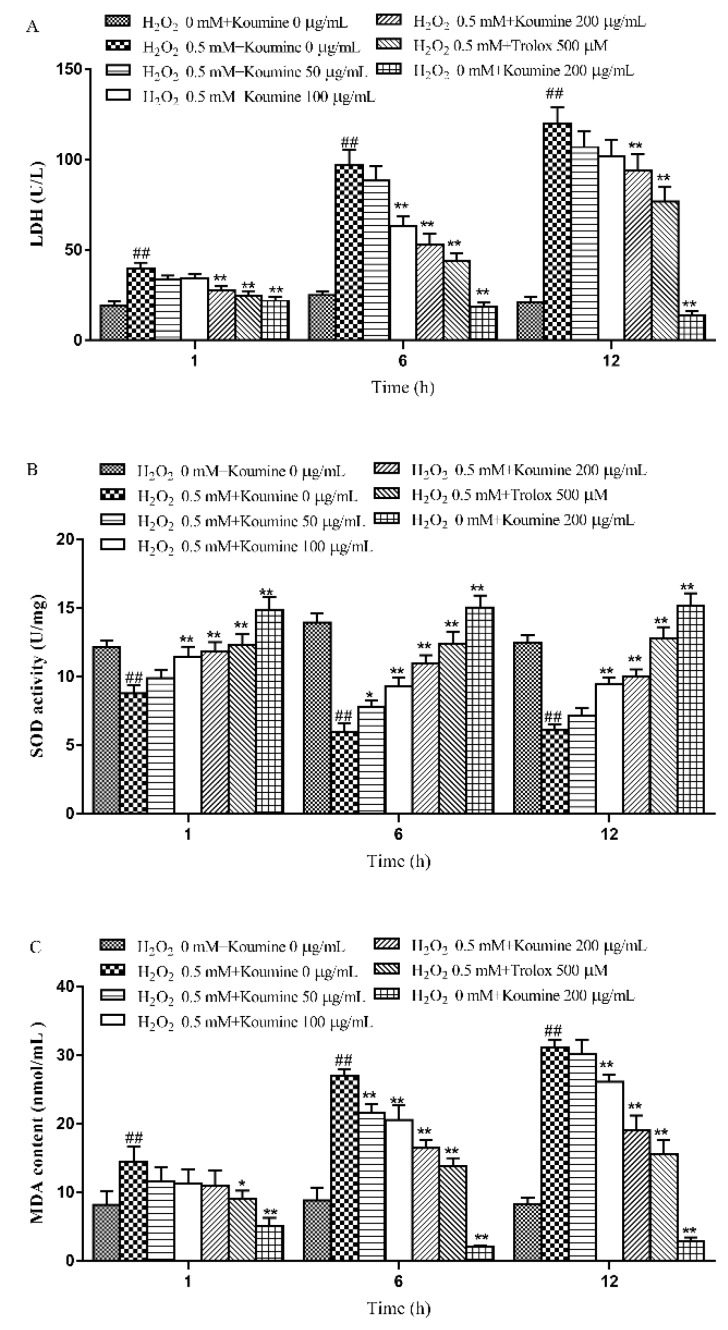

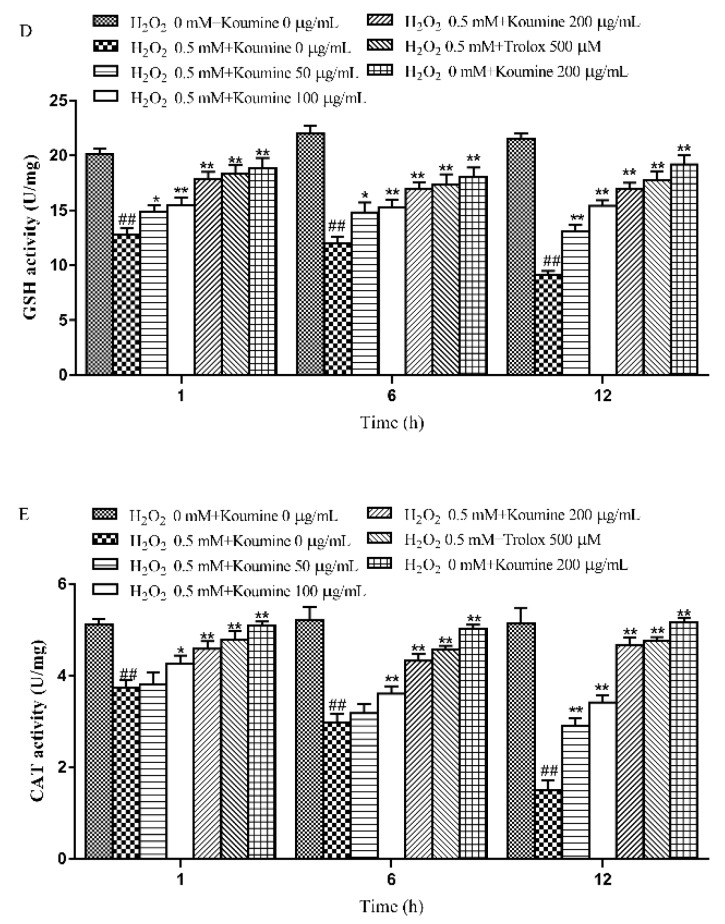
Effect of pretreatment of koumine on LDH activity (**A**), SOD activity (**B**), MDA content (**C**), GSH activity (**D**) and CAT activity (**E**) in H_2_O_2_-treated IPEC-J2 cells (mean ± s.d., *n* = 5). Legend: ## indicate level of significance at *p* < 0.01, respectively, compared with the control group; * and ** indicate level of significance at *p* < 0.05 and *p* < 0.01, respectively, compared with the oxidative stress model group.

The LDH activity in the cell culture supernatants was examined. A higher LDH activity reflects a higher rate of LDH leakage from the cells. Therefore, LDH activity can be used to evaluate the severity of cell damage. Compared with the control group, the rate of LDH leakage from IPEC-J2 cells increased significantly after exposure of the cells to H_2_O_2_ for 1, 6 and 12 h (*p* < 0.01). Compared with the model group, koumine (100 and 200 μg/mL) significantly (*p* < 0.05) reduced the rate of LDH leakage from IPEC-J2 cells that were exposed to H_2_O_2_ for 6 and 12 h, especially koumine at the dosage of 200 μg/mL koumine, in a dose-dependent manner. Meanwhile, there were no significant change at the dosage of 50 μg/mL (koumine) after exposure of the cells to H_2_O_2_ for 1, 6 and 12 h. The results are shown in Figure 4A.

Compared with the control group, SOD, GSH and CAT activities were markedly reduced, while the MDA content was significantly increased in IPEC-J2 cells treated with H_2_O_2_ for 1, 6 and 12 h (*p* < 0.01). Compared with the model group, koumine (100 and 200 μg/mL) significantly blocked the reduction in SOD activity, and the increase in MDA content induced by exposure to H_2_O_2_ for 1, 6 (*p* < 0.05) or 12 h (*p* < 0.01). Additionally, koumine (100 and 200 μg/mL) enhanced CAT and GSH activities. Furthermore, the activity of CAT were no significant differences at the dosage of 50 μg/mL (koumine) after exposure of the cells to H_2_O_2_ for 1 and 6 h. The results are shown in Figure 4B–E.

### 2.5. The Effect of Koumine on ROS Release from IPEC-J2 Cells with H_2_O_2_-Induced Damage

**Figure 5:**
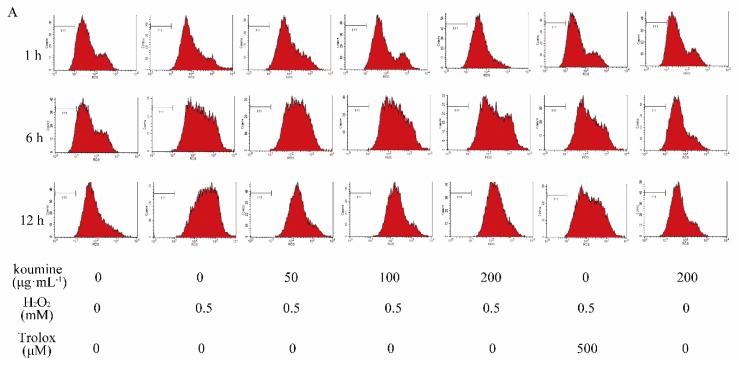

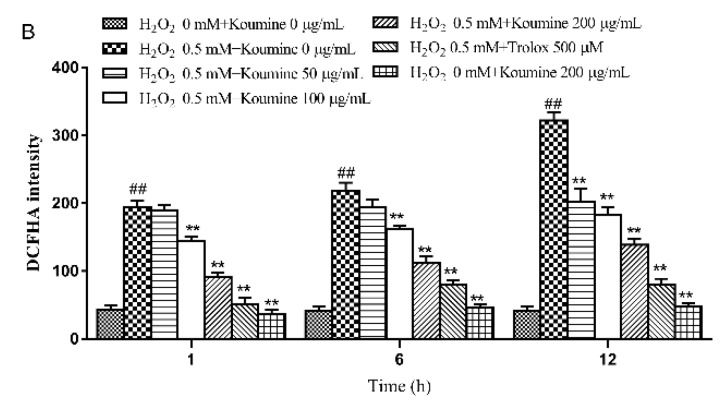
Effect of koumine on H_2_O_2_-induced release of ROS in IPEC-J2 cells (mean ± s.d., *n* = 5) Legend: ## indicate level of significance at *p* < 0.01, respectively, compared with the control group; ** indicate level of significance at *p* < 0.01, respectively, compared with the oxidative stress model group. (**A**) Intracellular ROS level was stained with DCFH-DA and detected by flow cytometry; (**B**) Quantified expression of ROS in the IPEC-J2 cells.

As shown in Figure 5, the amount of ROS released from the cells was significantly higher in the model group than in the control group at various time periods (*p* < 0.01). Compared with the model group, ROS release was reduced in the koumine (100 and 200 μg/mL) treated group in a dose-dependent manner. While there were no improvements in complication rate, there is a slow decline at the dosage of 50 μg/mL (koumine) after exposure of the cells to H_2_O_2_ for 1 and 6 h. The reduction in ROS release was highly significant (*p* < 0.01).

### 2.6. Mitochondrial Injury and Cell Apoptosis

**Figure 6:**
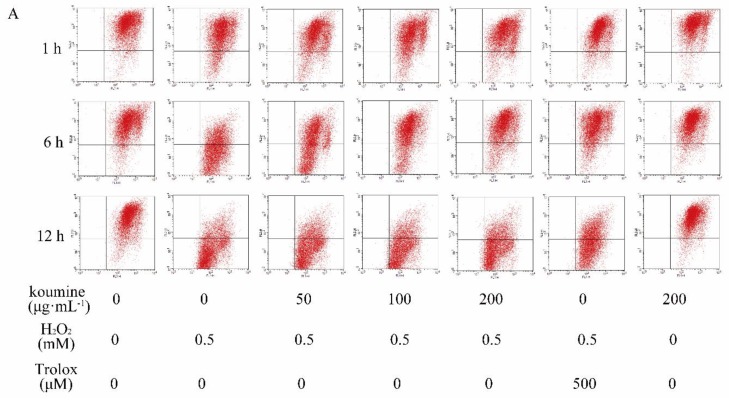

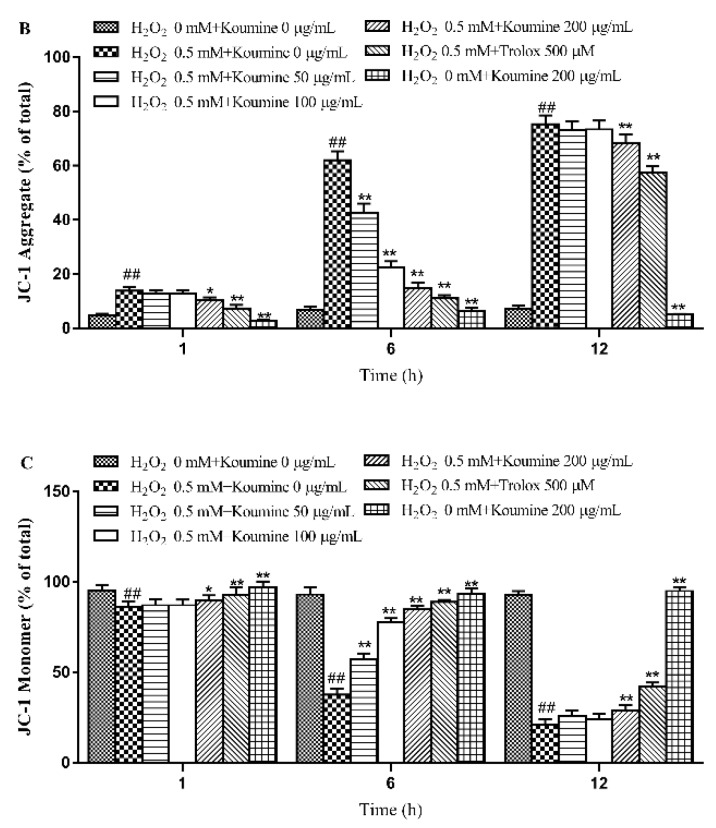
Effects of koumine on mitochondrial membrane potential in H_2_O_2_-induced IPEC-J2 cells. The mitochondrial membrane potential was measured using the JC-1 probe by flow cytometry. The data were expressed as mean ± s.d. (*n* = 3). ## *p* < 0.01 vs. control group; ** *p* < 0.01 vs. H_2_O_2_ group. (**A**) MMP was measured using the JC-1 probe by flow cytometry.; (**B**,**C**) JC-1 aggregates (cells emitting red fluorescence in the FL2 channel) suggested normal MMP in IPEC-J2 cells. In contrast, accumulation of JC-1 monomers monomers were respectively shown in the histograms.

**Figure 7:**
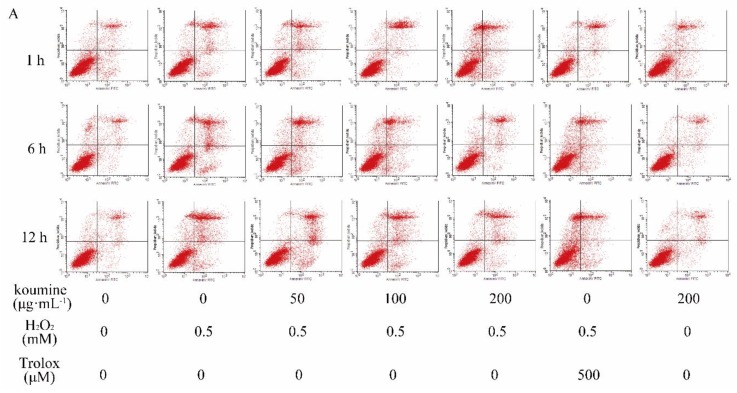

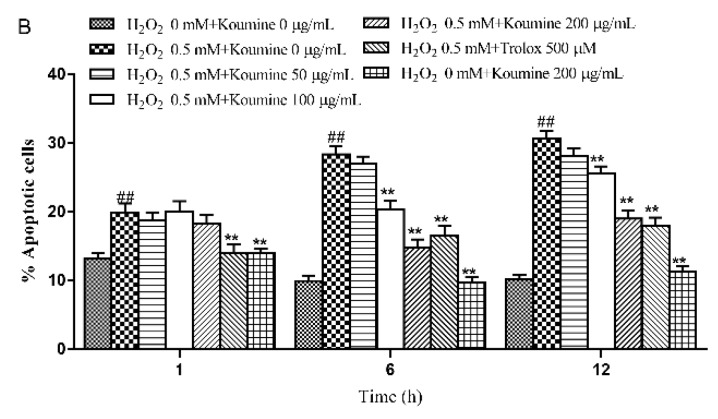
Effect of koumine on H_2_O_2_-induced apoptosis in IPEC-J2 cells (mean ± s.d., *n* = 5) Legend: # and ## indicate level of significance at *p* < 0.05 and *p* < 0.01, respectively, compared with the control group; * and ** indicate level of significance at *p* < 0.05 and *p* < 0.01, respectively, compared with the oxidative stress model group. (**A**) Effects of koumine treatment on H_2_O_2_-induced IPEC-J2 cellular apoptosis assayed by flow cytometry analysis of Annexin V-FITC/PI; (**B**) Quantification of the rate of apoptotic cells detected wit flow cytometry.

MMP depolarization, as reflected by the reduction of JC-1 aggregates and the accumulation of JC-1 monomers, usually occurs in apoptotic cells. Our results displayed that H_2_O_2_ treatment resulted in the conversion of JC-1 aggregates into JC-1 monomers as compared with untreated control cells, suggesting that MMP depolarization was induced by H_2_O_2_. However, this alternation was markedly reversed after koumine pretreatment, as reflected by increased JC-1 aggregates and reduced JC-1 monomers, indicating that koumine ameliorated H_2_O_2_-induced mitochondrial dysfunction in IPEC-J2 cells (Figure 6). To confirm cellular apoptotic rate, the apoptotic rates were measured by flow cytometry. The results indicated that compared with the control group, H_2_O_2_ exhibited a significant higher rate of apoptosis at 1, 6, 12 h (*p* < 0.01). Compared with H_2_O_2_ treatment, the apoptotic rate was dose-dependently reduced in koumine (100 and 200 μg/mL)-treated group (Figure 7). The reduction in the apoptotic rate was highly significant (*p* < 0.01). Furthermore, the apoptotic rate was no significant differences after exposure of the cells to H_2_O_2_ for 1, 6 and 12 h, but there is a slow decrease at the dosage of 50 μg/mL koumine.

### 2.7. Analysis of Caspase Activities

**Figure 8:**
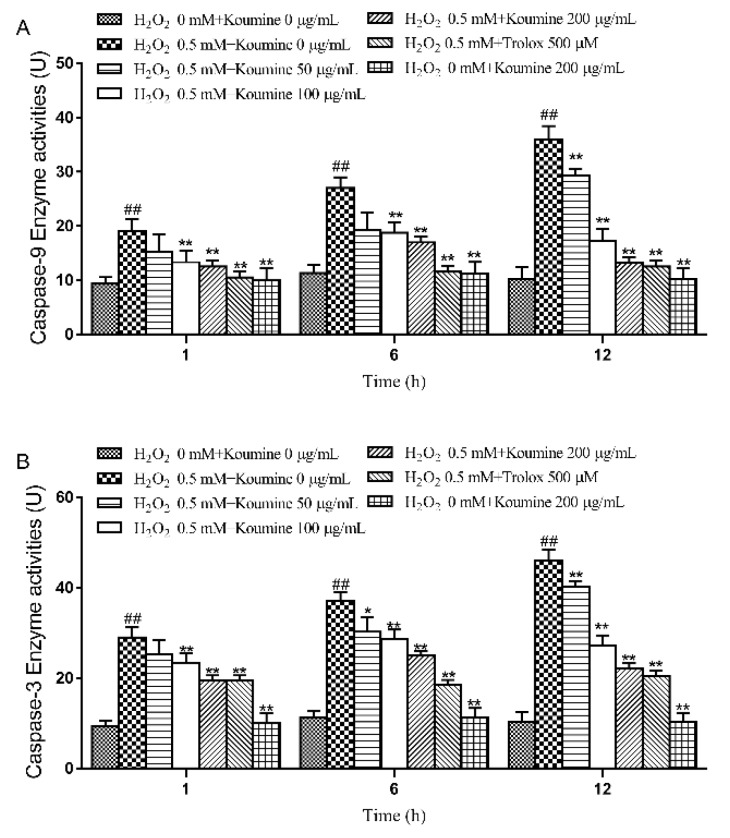
Effects of koumine on caspase activities in H_2_O_2_-induced IPEC-J2 cells. Cells were pretreated with 50–200 μg/mL koumine or 500 μM Trolox for 12 h, and then treated with 0.5 μM H_2_O_2_ for 1, 6 and 12 h in the preincubation mediums. Caspase activities were measured by caspase-3 and -9 activity assay kits. The data were expressed as mean ± SD (*n* = 3). ## *p* < 0.01 vs. control group; * *p* < 0.05 and ** *p* < 0.01 vs. H_2_O_2_ group. (**A**) The enzyme activities of caspase-9; (**B**) The enzyme activities of caspase-3.

As shown in Figure 8, our results showed that caspase-9 and -3 activities were significantly higher in H_2_O_2_-stimulated cells compared to control cells at 1, 6 and 12 h time points. Compared to H_2_O_2_-induced cells, koumine pretreatment dose-dependently inhibited caspase-9 and -3 activities mediated by H_2_O_2_ at 1, 6 and 12 h time points. 

### 2.8. Regulation of Apoptosis-Related Proteins

**Figure 9:**
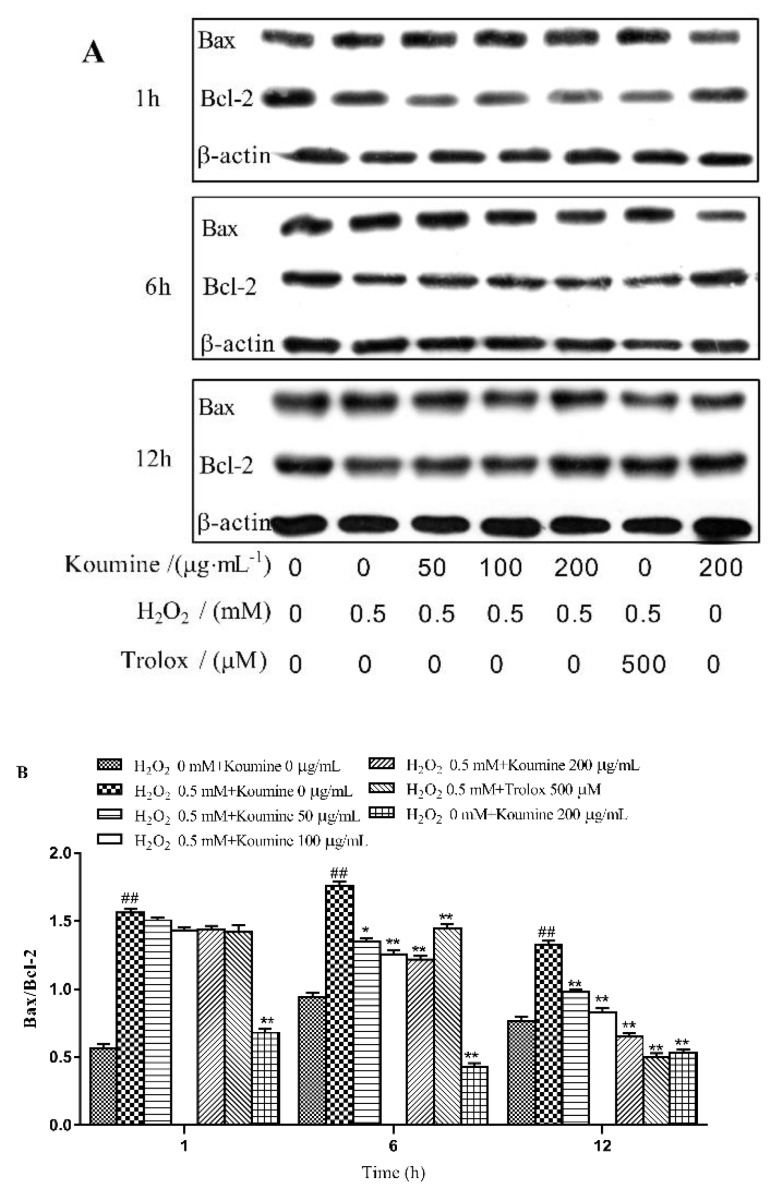
Effect of koumine on Bcl-2 and Bax protein expression in H_2_O_2_-treated IPEC-J2 cells (mean ± s.d., *n* = 5). The data were expressed as mean ± s.d. (*n* = 3). ## *p*< 0.01 vs. control group; * *p* < 0.05 and ** *p* < 0.01 vs. H_2_O_2_ group. (**A**) The expressions of Bax and Bcl-2 proteins; (**B**) The ratio of Bax / Bcl-2 protein level.

Compared with the control group (Figure 9), the Bax/Bcl-2 ratio was significantly higher after exposure of the cells to H_2_O_2_ for 1, 6 and 12 h (*p* < 0.01). Koumine inhibited the H_2_O_2_-induced reduction in Bcl-2 protein expression level and the H_2_O_2_-induced increase in Bax protein expression level in a dose-dependent manner, resulting in decreased Bax/Bcl-2 ratios (1 h, *p* < 0.05; 6 and 12 h, *p* < 0.01).

## 3. Discussion

In our study, IPEC-J2 cells were used as a cellular model to investigate the effects of koumine on the antioxidant defense systems. We had shown that H_2_O_2_ markedly decreased the viability of IPEC-J2 cells, whereas pretreatment with koumine significantly inhibited cell injury, as demonstrated by MTT assay. We also detected the activities of LDH and SOD, content of MDA, with and without H_2_O_2_. The results indicated that H_2_O_2_ could induce IPEC-J2 cells damage, which led to the increase of LDH and MDA and reduction of SOD activity. Oxidative stress caused by ROS is responsible for a wide variety of cellular damage and is the most validated mechanism of secondary injury [13]. Following oxidative stress, the overproduction of ROS and subsequently the depletion of antioxidants resulted in the total breakdown of the endogenous antioxidant defense mechanisms, culminating in failure to protect cells from oxidative damage. Among the antioxidant enzymes and proteins are SOD, GSH and CAT that catalyze the processing of ROS to less toxic molecules. GSH and CAT are some of the main antioxidant defenses in the cells [14]. Among biomarkers of oxidative stress, MDA and SOD are known to be two sensitive indicators [15]. MDA is the end product of lipid peroxidation and MDA levels reflect the extent of cell damage due to oxidative stress [16]. ROS including hydrogen peroxide (H_2_O_2_), hydroxyl radical (OH^−^) and superoxide anion (O^2−^) are physiologically produced at a basal rate. H_2_O_2_ may induce the generation of ROS at mitochondria which has been widely used as a model exogenous oxidative stress mediated experiment in intestinal epithelium apoptosis [17,18]. Koumine pretreatment effectively protected IPEC-J2 cells from H_2_O_2_-induced damage. Koumine obviously reduced ROS release, increased the activities of SOD, CAT and GSH, decreased the level of LDH and the content of MDA, and inhibited the apoptosis induced by H_2_O_2_.

Apoptosis, the type I form of programmed cell death, is induced by various intracellular stimuli, including DNA damage, bacteria invasion and oxidative stress, via outer mitochondrial membrane permeabilization [19]. Mitochondria, unique organelles for energy transformation, are particularly vulnerable to oxidative stress after mitochondrial dysfunction of mitochondrial membrane potential. The mitochondria respiratory chain is a major site of ROS production in the cells, and mitochondria are crucial for cellular proliferation, cell apoptosis, as well as regulation of the cellular redox state [20]. Caspases are a group of aspartate specific cysteine protease, which plays a key role in regulating the apoptosis induced by different kind of stimuli including oxidative stress. Functionally, caspase-3 is an important effector in the apoptotic process, and caspase-9 is an initiator of caspase-3 in the mitochondria-dependent pathway [21]. Mitochondria are crucial for regulation of the cellular redox state, cell apoptosis, as well as cellular proliferation. It is particularly vulnerable to oxidative stress, and mitochondria respiratory chain is a major site of ROS production in the cells. The expressions of pro- and anti-apoptotic Bcl-2 family proteins which belong to intrinsic mitochondrial apoptotic pathway are changed. Subsequently, various caspases are stimulated, and cell apoptosis is irreversible, indicating that ROS as mediators produced by the mitochondria appears to be associated with early and late steps of the regulation of cell apoptosis [20,22]. Some studies indicate that the anti-apoptotic Bcl-2 family proteins prevent activations of caspases at their upstream to prevent apoptosis [23]. It has also been found that suppression of apoptosis through anti-apoptotic Bcl-2 and Bcl-xL is relative to a reduction of the cellular redox potential and/or a protection against ROS [24]. In this study, we determined that H_2_O_2_ significantly increased IPEC-J2 cell apoptosis. We also found that H_2_O_2_ decreased mitochondrial membrane potential, promoted activations of caspase-3 and caspase-9, increased expressions of Bax and decreased level of Bcl-2 protein in IPEC-J2 cells, suggesting that H_2_O_2_ likely induced apoptosis through caspase-dependent mitochondrial death pathway. However, koumine pretreatment reversed this effect. The data showed that IPEC-J2 cells pretreated with koumine suppressed H_2_O_2_-stimulated mitochondrial apoptotic pathway including structural mitochondrial damage, decrease of expression of Bcl-2 protein and activation of caspase-9 and -3, increase of expressions of Bax proteins.

In summary, the present study shows that H_2_O_2_ can induce IPEC-J2 cells injury and induce cells apoptosis. Koumine protects IPEC-J2 cells against H_2_O_2_-induced oxidative stress, cells apoptosis, ROS activity, and activities of caspase-9 and caspase-3. Koumine also can regulate the expression of Bcl-2 and Bax. These data help explain the protective action of koumine against cell injuries involving the mitochondrial pathway and provides a theoretical basis for the further development and application of koumine.

## 4. Materials and Methods

### 4.1. Materials

Porcine intestinal epithelial cells (IPEC-J2) were purchased from the National Type Culture Collection (NTCC)-Biovector. Fetal bovine serum (FBS) and Dulbecco’s Modified Eagle Medium/Nutrient Mixture F-12 (DMEM/F-12) were purchased from Gibco. The Annexin V-FITC Apoptosis Detection Kit and BCA Protein Assay Kit were purchased from Beyotime Biotechnology Co., Ltd. (Jiangsu, China). The fluorescent probe 2’, 7’-dichlorodihydrofluorescein diacetate (DCFH-DA) was acquired from SolarBio Science & Technology Co., Ltd. (Shanghai, China). The lactate dehydrogenase (LDH), superoxide dismutase (SOD), catalase (CAT), glutathione (GSH) and malondialdehyde (MDA) assay kits were purchased from Nanjing Jiancheng Bioengineering Institute (Nanjing, China). Caspase-3 and -9 activity assay kits were purchased from Beyotime (Nanjing, Jiangsu, China). Antibodies for B-cell lymphoma 2 (Bcl-2), Bcl-2-associated X (Bax) and β-actin were purchased from Proteintech (USA). The anti-Bax, anti-Bcl-2 and anti-β-actin antibodies were diluted 1:5000, 1:5000 and 1:4000, respectively. Polyvinylidene fluoride (PVDF) membrane and Amersham ECL Advance Western blot detection kit were obtained from GE Healthcare Life Science (Piscataway, NJ, USA).

### 4.2. Cell Culture

IPEC-J2 cells were grown and maintained in complete medium, which consisted of a 1:1 mixture of Dulbecco’s modified Eagle’s medium and Ham’s F-12 Nutrient Mixure (DMEM/F12, plain medium) supplemented with 5% fetal bovine serum, 5 μg/mL insulin, 5 μg/mL transferrin, 5 ng/mL selenium, 5 ng/mL epidermal growth factor and 1% penicillin-streptomycin. Cells were grown at 37 °C in a humidified atmosphere of 5% CO_2_.

### 4.3. Assessment of Cell Cytotoxicity

To select the optimal H_2_O_2_ concentration, IPEC-J2 cells were seeded into 96-well plates, as described above. Then, H_2_O_2_ at different concentrations (0.5, 1, 2 and 4 mM) were added in the wells and incubated for 1, 6 and 12 h. The negative controls were treated with PBS instead of H_2_O_2_. Cells were twice washed with PBS to remove the H_2_O_2_ and cell activity was determined by MTT reduction assay. MTT (the final concentration of 0.5 mg/mL) was added for 4 h. Subsequently, dimethyl sulfoxide (DMSO) was added to dissolve the formazan crystals after removing the culture medium. Data was read immediately at 570 nm. The results are expressed as the percentage of reduced MTT, assuming the absorbance of control cells to be 100%. Samples were measured in quintuplicate, and the experiment was repeated three times.

For cell cytotoxic assay, IPEC-J2 cells were seeded on 96-well plates, as described above. Then, koumine at different concentrations (50, 100 and 200 μg/mL) was added. After incubating for 12 h, 0.5 mM H_2_O_2_ was added to each well. After incubation for 1, 6 and 12 h, the cell activity was tested with the MTT assay. Samples were measured in quintuplicate, and the experiment was repeated three times.

### 4.4. Enzyme Assays

The cellular activities of LDH, CAT, GSH and SOD and the MDA content were examined using commercial kits and performed following the manufacturers’ instructions. The results were read on a multi-functional fluorescence microplate reader.

### 4.5. Examination of the ROS Production and Released from Cells

The ROS Detection Kit S0033 (Beyotime Biotechnology Co., Ltd.) was used. DCFH-DA was diluted 1:1000 in serum-free medium to a final concentration of 10 μmol/L. After removal of the culture medium, the cells were detached, centrifuged (1000× *g*, 5–10 min) and collected. Subsequently, the cells were mixed with 1 mL of DCFH-DA working solution and incubated for 20 min in a 37 °C incubator. The cells were then washed twice with PBS and centrifuged at 1000× *g* for 5 min. The pellets were collected and examined by fluorescence flow cytometry.

### 4.6. MMP Measurements and Examination of Apoptosis

MMP in IPEC-J2 cells was detected using a dual emission potential-sensitive fluorescent probe JC-1 (Beyotime Biotech, Nantong, China) according to the manufacturer’s instructions. Briefly, IPEC-J2 cells initially were rinsed twice in PBS and incubated with JC-1 for 20 min at 37 °C. Then, the cells were rinsed with PBS again and analyzed with fluorescence flow cytometry.

Apoptosis was examined using the Annexin V-FITC/PI Apoptosis Detection Kit following the manufacturer’s instruction. Cells from various treatment groups were detached, centrifuged at 1000 r/min for 5 min and collected. After removal of the supernatants, the cells were washed with PBS. The supernatants were discarded, and the cell pellets were resuspended in the binding buffer. Subsequently, the cells were mixed gently with 1.25 μL of Annexin V-fluorescein isothiocyanate (FITC) and incubated for 10 min in the dark. Cells that had not been exposed to Annexin V-FITC were used as negative control. Following Annexin V-FITC labeling, the cells were examined by flow cytometry.

### 4.7. Caspase-3 and -9 Activities

The Caspase-3 and Caspase-9 Activity Kits (Beyotime, Nanjing, China) were used to measure the activities of caspase-3 and -9, which is based on spectophotometric detection of the chromophore *p*-nitroaniline (*p*NA) after cleavage from the labeled substrate acetyl-Asp-Glu-Val-Asp *p*-nitroanilide (Ac-DEVD-*p*NA) and acetyl-Leu-Glu-His-Asp *p*-nitroanilide (Ac-LEHD-*p*NA), respectively. Cells were seeded on 96-well plates, as described above. Then, koumine at different concentrations (50, 100 and 200 μg/mL) was added. After incubating for 12 h, 0.5 mM H_2_O_2_ was added to each well for 1, 6 and 12 h. For assays, 1 × 10^6^ cells were washed with cold PBS, lysed on ice. After centrifugation 16,000–20,000× *g* for 15 min at 4 °C. Caspase assays performed in 96-well plates by incubating 50 μL supernatant per sample with 10 μL caspase substrate (Ac-DEVD-*p*NA or Ac-LEHD-*p*NA) (2 mM) and 40 μL reaction buffer (1% NP-40, 20 mM Tris–HCl (pH 7.5), 137 mM NaCl and 10% glycerol) for 1.5 h at 37 °C. Absorbance was read at 405 nm using a microplate reader (MK3, Thermo, Waltham, MA, USA). Caspase-9 and Caspase-3 activity was expressed as the change in enzyme activity relative to untreated control cultures.

### 4.8. Examination of Bax, Bcl-2 and β-actin Expression by Western Blotting

The cells were pretreated with various concentrations of koumine for 12 h. Cells treated with Trolox were used as positive controls. The cells were then incubated in medium containing H_2_O_2_ for 1, 6, and 12 h. After incubation, total cellular protein was extracted and quantified using the bicinchoninic acid (BCA) assay. Polyacrylamide gels (10%) were prepared. Protein samples were subjected to sodium dodecyl sulfate-polyacrylamide gel electrophoresis (SDS-PAGE) and transferred to polyvinylidene fluoride (PVDF) membranes. The expression of Bax, Bcl-2 and β-actin proteins was analyzed by Western blotting. The results were analyzed using the Bio-Rad Gel Imaging System. Protein expression levels were expressed as OD values. Beta-actin was used as an internal control.

### 4.9. Statistical Analysis

The data were statistically analyzed using SPSS19.0 software. The results are expressed as the mean ± standard deviation (s.d.). A p value less than 0.05 indicates that the difference was statistically significant.
